# Determination of Ivermectin in Medicated Feeds by Liquid Chromatography with Fluorescence Detection

**DOI:** 10.1155/2013/362453

**Published:** 2013-12-19

**Authors:** Konrad Pietruk, Piotr Jedziniak

**Affiliations:** Department of Pharmacology and Toxicology, National Veterinary Research Institute, 57 Partyzantow Avenue, 24-100 Pulawy, Poland

## Abstract

A labour- and time-effective analytical procedure for determination of ivermectin in medicated feed at recommended level of 2.0 mg kg^−1^ has been developed and validated. The analyte was extracted from grinded feed samples with acetonitrile and derivatisated with N-methylimidazole and trifluoracetic anhydride. The fluorescent derivatives were analysed by liquid chromatography method using C8 column. The isocratic conditions using acetonitrile, methanol, water, and tetrahydrofuran were applied. Fluorescence detection was performed at 365 nm (excitation) and 475 nm (emission) wavelengths. The total analysis time was 10 min. The validation results of the method (within-laboratory reproducibility 4.0% CV, mean recovery 100.1%) confirm the appropriate precision and accuracy of the developed method.

## 1. Introduction

Ivermectin is a macrocyclic lactone that has been known as an antiparasitic agent for almost 30 years [1–3]. It is a veterinary medicine with a broad spectrum of action, showing very high effectiveness against gastrointestinal and pulmonary nematodes, as well as for gadflies and mites. Because of its potency, ivermectin is considered to be an effective antiparasitic agent for cattle, pigs, and sheep [4, 5].

Ivermectin can be administrated throughout many routes, to name a few: injection, medicated feed, pills, and pour-on applications. One of the most popular ways of the administration of ivermectin to animals is the form of medicated feed, which consists of ivermectin premix and suitable feed. The advantages of medicated feed are high effectiveness, ease of administration, and high tolerance by the animals [6, 7]. The important factor to consider in the production of medicated feeds is recommended concentration for a specific animal (2.0–2.4 mg kg^−1^ in feed for pigs).

The usage of pharmacologically active substances in animal feeds requires detailed control to assure safety of administered drugs. Both underdosage and overdosage of the active substance carry consequences. The overdose can lead to longer persistence of drug residues in food of animal origin or even poisonings of animals, while too low concentration is associated with the lack of therapeutic effect and the possibility of growing of resistance. Therefore, there is a need to control the content of active substances in feeds (compliance with declaration) and to confirm homogeneity of medicated feeds using appropriate methods [8, 9].

Wide spectrum of methods for the determination of ivermectin in biological material, based on liquid chromatography with UV, FLD and, MS/MS detection, has been developed [10–16]. However, only a few authors have published methods of the determination of ivermectin in animal feeds [17–19]. The procedure presented by Doherty et al. is based upon liquid chromatographic analysis with reverse-phase C18 column and ultraviolet detection. In this method, ivermectin was extracted from feed using methanol, and then the analyte was separated from matrix interferences by solid-phase extraction (SPE) clean up step using Sep-Pak C18 and silica cartridges. Very recently, the method for determination of six macrocyclic lactones in food and feed by Galarini et al. has been developed. In this method, fluorescence detection after derivatisation of analytes with N-methylimidazole, trifluoracetic anhydride, and acetic acid is used [20].

The purpose of this study was to develop a method, applicable in the determination of ivermectin in feed at recommended concentrations for pigs, based on the HPLC-FLD detection after precolumn derivatisation [21].

## 2. Material and Methods

### 2.1. Chemicals and Standards

Analytical standards used during method development were purchased from the following manufactures: doramectin (internal standard, IS from Riedel-de Haën (Germany) and ivermectin from Sigma (Germany).

Acetonitrile (ACN) and methanol, both HPLC grade, were purchased from J.T. Baker (Germany). N-methylimidazole, trifluoroacetic anhydride, and tetrahydrofuran (THF) were bought from Sigma-Aldrich (Germany). Ultrapure water (resistance > 18 mΩ) was produced by Milli-Q system (Millipore, France).

### 2.2. Preparation of Standard Solutions

Stock standard solutions (1000 *μ*g ml^−1^) were prepared by weighing of 10 mg of each analytical standard and dissolving in 10 mL of methanol. They were stable during 12 months when stored in 2–10°C. Working standard solutions (100 *μ*g mL^−1^ and 10 *μ*g mL^−1^) were prepared by the dilution of the proper quantity of stock standard solutions with methanol and were stored in 2–10°C (stable for 6 months).

### 2.3. Equipment

In the sample preparation, vortex mixer (IKA, Germany), laboratory shaker (Barnstead, USA), laboratory grinder (Glen Mills, USA), analytical balance (Mettler Toledo, USA), laboratory centrifuge (Sigma, Germany) and nitrogen evaporation system (VLM, Germany) were used. The analysis was performed using Varian ProStar liquid chromatography system (Varian, USA) consisting of Solvent Delivery Module model 240, autosampler model 410, column oven model 510, and fluorescence detector (FLD) and controlled by Galaxie 1.9 software and Phenomenex Luna C8 (2) column (150 mm, 4.6 mm, 3 *μ*m, Phenomenex, USA) with C8 precolumn.

### 2.4. Sample Preparation

The day before the analysis, 5 g ± 0.01 g of grinded feed was weighed into 50 mL polypropylene tube, fortified with 100 *μ*L of internal standard (IS, doramectin, 100 *μ*g ml^−1^) and well mixed using vortex mixer.

For quality assurance, the spiked control sample was prepared and analysed in each analytical batch. The day before the analysis, 5 g ± 0.01 g of grinded blank feed sample was fortified with 100 *μ*L of ivermectin (100 *μ*g mL^−1^) and 100 *μ*L of internal standard (100 *μ*g mL^−1^), and well mixed using vortex mixer.

25 mL of acetonitrile was added to each analysed sample; the sample was mixed, shaken (30 min, 200 cycles min^−1^), and then centrifuged (4500 ×g, 20°C, 15 min). The supernatant (1 mL) was transferred to the glass tube and evaporated to dryness (N_2_, 50°C). The derivatization step was performed by adding 100 *μ*L of ACN : N-methylimidazole solution (1 : 1, v : v) and 150 *μ*L of ACN : trifluoroacetic anhydride solution (1 : 2, v : v). The sample was well mixed and transferred into the vial.

### 2.5. Liquid Chromatography Conditions

The chromatographic separation was performed using Phenomenex Luna C8 column and mobile phase consisting of acetonitrile, methanol, water, and tetrahydrofuran (48 : 45 : 5 : 2, v : v : v : v) pumped in isocratic conditions. The flow rate of the mobile phase was 1 mL min^−1^. The column oven temperature was set at 40°C. The injection volume was 5 *μ*L. The fluorescence detection was performed at the wavelengths of 365 nm and 475 nm excitation and emission, respectively. The total run time of the instrumental analysis was 10 min.

### 2.6. Method Validation

The overall concept of validation studies (repeatability, trueness) was based on the guideline on validation of analytical procedures by The European Agency of the Evaluation of Medicinal Products [22]. According to the authors' knowledge, no validation guideline was published, designed exclusively for medicated feed.

The linearity of the FLD detector response to ivermectin was verified in the range of 0.4–4.0 *μ*g ml^−1^. The study was performed by preparing three separate curves on five levels of ivermectin (0.4, 0.8, 1.6, 2.4, and 4.0 *μ*g mL^−1^) with doramectin as an IS in mobile phase.

A working range of the described method was verified by preparing matrix calibration curve. The ivermectin was determined in three series of blank feed fortified with ivermectin at specific concentration range, namely, 0.5, 1.0, 2.0, 3.0, and 5.0 mg kg^−1^.

Linearity and working ranges were calculated by injecting five solutions at increasing concentrations. Calibration curves were obtained by least-squares linear regression analysis of the peak area ratio (ivermectin to doramectin) versus concentration.

Repeatability and within-laboratory reproducibility studies were performed by the fortification of six blank feed samples with ivermectin at a concentration of 2 mg kg^−1^ and analysed in the three following days by the developed procedure.

Recovery was calculated as a percentage of the true concentration of a substance recovered during the analytical procedure.

The trueness of the method was considered acceptable when the measured values for the relative recovery rate were in the range of 70–110% [22].

The limit of detection (LOD) of ivermectin was calculated as 3 times of standard deviation of signal response (noise) of 10 blank feeds. The lowest concentration of matrix calibration curve was regarded as the limit of quantification (LOQ).

The developed method was used to perform homogeneity tests. Four feed samples with ivermectin were analysed using the presented method.

### 2.7. Statistical Evaluation of Validation Results

The ANOVA was used to calculate standard deviation and coefficient of variation in repeatability and reproducibility studies.

The HorRat test was used to calculate the ratio of the experimentally obtained standard deviation of within-laboratory reproducibility and the target standard deviation calculated using Horwitz equation [23, 24]:

(1) $$\begin{array}{c} \partial =2^{\left(1-0.5\log C\right)}, \end{array}$$

where *C* is the mass fraction expressed as a power of 10.

The original data developed from interlaboratory (among-laboratory) studies assigned a HorRat value of 1.0 with limits of acceptability of 0.5 to 2.0. Based on the experience and for the purpose of exploring the extrapolation of HorRat values to repeatability studies, taking the minimum of acceptability one-half of the lower limit (0.5 × 0.5 ≈ 0.3) and the maximum acceptability two-third of the upper limit (0.67 × 2.0 ≈ 1.3); thus, acceptable HorRat values were 0.3–1.3 [25].

## 3. Results and Discussion

### 3.1. HPLC Conditions

**Figure 1:**
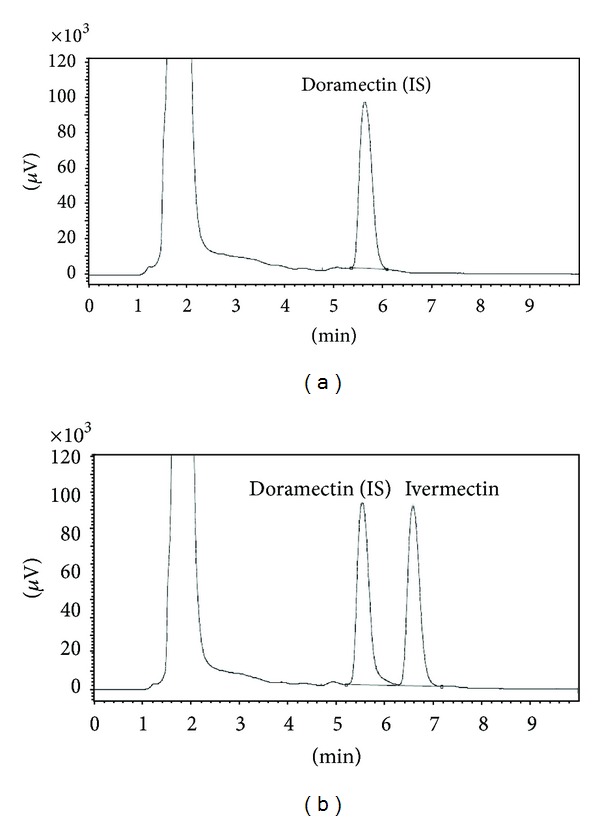
Chromatograms of blank feed (a) and feed fortified with ivermectin at concentration of 2 mg kg^−1^ (b).

Chromatograms of the blank animal feed sample (Figure 1(a)) and the feed sample fortified with ivermectin at concentration of 2 mg kg^−1^  (Figure 1(b)) show that there are no interfering peaks and that sensitivity is acceptable for determination of an analyte and the internal standard.

The developed method has been proven to be quicker compared to the method developed by Galarini et al. [20]. The total run time of the presented method is 10 min where retention time of ivermectin is about 6.75 min compared to the total run of 14 min and ivermectin retention time about 9 min reported in the method of those authors [20].

The isocratic conditions applied allow to avoid time consumption necessary for column equilibration in the methods using gradient programs for mobile phase [20]. That makes the presented method faster, allows to analyse higher number of samples in single work day, and minimalizes the consumption of energy, solvents, and reagents.

The presented method is focused on determination of ivermectin in medicated feed, which can be considered as a potential disadvantage. The authors decided to develop the method only for ivermectin because it is the most popular antiparasitic agent used in medicated feed for pigs. Nevertheless, the developed method can be extended for additional macrocyclic lactones.

### 3.2. Sample Preparation

In the described method, grinded feed sample was treated with acetonitrile as an extraction agent; it allows to obtain similar recovery as in the method of other authors [20] who used acetone for extraction of ivermectin from medicated feed. Thanks to high specificity of fluorescence detection, no clean-up step after extraction was required. The derivatisation procedure applied in the presented method was earlier successfully applied in determination of macrocyclic lactones in animal tissues [21]. The reaction of derivatisation was almost instantaneous (30 s) and required less reagents compared to the method developed by other authors [20]. Thanks to simple and fast sample preparation, up to 30 samples per analytical run can be done using the presented method during a routine workday.

### 3.3. Validation Results

Standard calibration curve and matrix calibration curve were found to be linear and correlation coefficient in the both cases was 0.99. The validation of the method proved its applicability in the determination of ivermectin in feed in the range of 0.5–5 mg kg^−1^. The LOD and LOQ values (0.03 mg kg^−1^ and 0.5 mg kg^−1^, resp.) show sufficient sensitivity for routine analysis.

The mean concentrations and standard deviations calculated in repeatability and within-laboratory reproducibility confirm adequacy of the developed method for the determination of ivermectin at the recommended concentration (2 mg kg^−1^). The calculated CV of the method (3.0% for repeatability and 4.0% for within-laboratory reproducibility) was in all cases below 8.0% which is a target CV according to Guidelines for Standard Method Performance Requirements that confirm appropriate precision of the developed method at this level [25].

The trueness of the method was confirmed with recovery rate close to 100%.

Calculated HorRat value (0.38) is in the range between 0.3 and 1.3 and confirms a success of validation process. Calculated in ANOVA, studies, *P* value is greater than significance level, which means that there is no statistically significant difference between groups of data. That proves that the method is robust and the results of analysis are independent despite technicians engaged in the analysis, different batches of reagents used in the analysis, and the day when the analysis was performed.

The validation results (Table 1) have revealed that the method used to extract, derivatise, and quantify ivermectin by FLD detection enabled obtaining reliable results. Validation parameters of the method presented are not worse than these obtained by the methods involving other analytical techniques [17–20].

### 3.4. Application to Real-Life Samples

**Figure 2:**
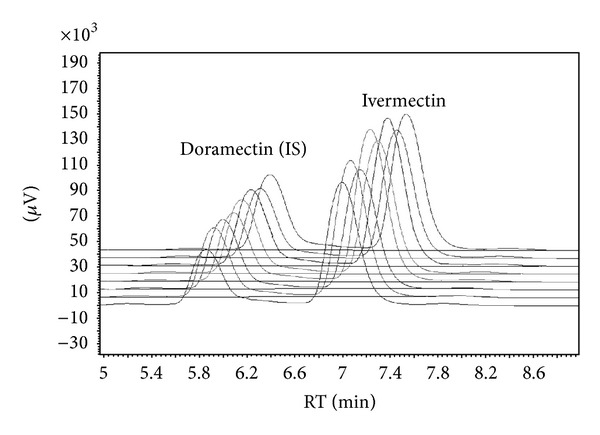
Chromatograms of animal feed samples analysed in homogeneity studies. Mean concentration: 3.87 mg kg^−1^, standard deviation: 0.19 mg kg^−1^, and CV: 4.8%.

The developed method was used in the laboratory to commercially perform tests for homogeneity of medicated feeds containing ivermectin.  Figure 2 presents example of chromatograms of homogeneity studies. In this study, each of the four samples was analysed twice and coefficient of variation calculated was 4.8%.

## 4. Conclusions

The method presented in this paper confirms the adequacy of postcolumn derivatisation combined with fluorescence detection as a valid approach to determination of macrocyclic lactones in feed. The method is simple, fast, and relatively cheap, due to the lack of clean-up step and good efficacy of derivatisation. That also leads to high sample throughput in a single workday; validation results confirm that the method fits to the purpose for determination of ivermectin in medicated feeds at recommended concentration. The method was already successfully applied in the commercially performed homogeneity studies that also prove its usefulness.

## Figures and Tables

**Table 1:** Validation results.

	Day 1	Day 2	Day 3
Standard calibration curve	*y* = 0.50*x* − 0.03, *R* ^2^ = 0.99		
Matrix calibration curve	*y* = 0.48*x* + 0.03, *R* ^2^ = 0.99		
Mean concentration (mg kg^−1^) (repeatability)	2.00	1.97	2.04
Standard deviation (mg kg^−1^) (repeatability)	0.06		
CV (%) (repeatability)	3.0		
HorRat (repeatability)	0.38		
Mean recovery (%) (repeatability)	100.1	98.4	101.8
Mean concentration (mg kg^−1^) (within-laboratory reproducibility)	2.00		
Standard deviation (mg kg^−1^) (within-laboratory reproducibility)	0.08		
CV (%) (within-laboratory reproducibility)	4.0		
Mean recovery (%) (within-laboratory reproducibility)	100.1		
LOD (mg kg^−1^)	0.03		
LOQ (mg kg^−1^)	0.50		
*P* value (ANOVA)	0.20		
